# An Efficient *In Vitro* Plantlet Regeneration from Shoot Tip Cultures of *Curculigo latifolia*, a Medicinal Plant

**DOI:** 10.1155/2014/275028

**Published:** 2014-02-27

**Authors:** Nahid Babaei, Nur Ashikin Psyquay Abdullah, Ghizan Saleh, Thohirah Lee Abdullah

**Affiliations:** ^1^Islamic Azad University, Bushehr Branch, 7519619555 Bushehr, Iran; ^2^Department of Crop Science, Faculty of Agriculture and Food Science, Universiti Putra Malaysia Bintulu Campus, Jalan Nyabau, 97008 Bintulu, Sarawak, Malaysia; ^3^Department of Crop Science, Faculty of Agriculture, Universiti Putra Malaysia UPM, 43400 Serdang, Selangor, Malaysia

## Abstract

A procedure was developed for *in vitro* propagation of *Curculigo latifolia* through shoot tip culture. Direct regeneration and indirect scalp induction of *Curculigo latifolia* were obtained from shoot tip grown on MS medium supplemented with different concentrations and combinations of thidiazuron and indole-3-butyric acid. Maximum response for direct regeneration in terms of percentage of explants producing shoot, shoot number, and shoot length was obtained on MS medium supplemented with combination of thidiazuron (0.5 mg L^−1^) and indole-3-butyric acid (0.25 mg L^−1^) after both 10 and 14 weeks of cultures. Indole-3-butyric acid in combination with thidiazuron exhibited a synergistic effect on shoot regeneration. The shoot tips were able to induce maximum scalp from basal end of explants on the medium with 2 mg L^−1^ thidiazuron. Cultures showed that shoot number, shoot length, and scalp size increased significantly after 14 weeks of culture. Transferring of the shoots onto the MS medium devoid of growth regulators resulted in the highest percentage of root induction and longer roots, while medium supplemented with 0.25 mg L^−1^ IBA produced more numbers of roots.

## 1. Introduction

The monocotyledonous plant, *Curculigo latifolia,* commonly known as lemba is a perennial herb belonging to the Hypoxidaceae family and was thought to be natively from Malaysia. The plant is known for its sweet proteins, namely, curculin and neoculin, that have been proven to be 500 to 9000 times sweeter than sucrose by weight [1–4]. Curculin exhibits both sweet tasting and taste modifying activities, which change a sour taste into a sweet taste [5–7] and is accumulated in fruit during ripening [8]. Curculin which is a good low-calorie sweetener is absorbed by the human body [7] and has a great potential for low-calorie sweetener-based industries. Besides the industrial and economic importance of *C. latifolia,* it is also considered as a valuable medicinal plant in having anticancer properties [9] and antidiabetic properties [10] and inhibiting hepatitis B virus [11]. This makes *C. latifolia* a desirable plant with great potential for the pharmaceutical industry [12].

*Curculigo* is found to be wild in the forest and also on road side. It is currently brought into cultivation by planting seedlings collected from the wild. Not only this is destructive but also it can cause declining of its population if without supervision [13] few issues are also needed to be put into serious consideration. Firstly, genetic variations found amongst accessions used as planting materials can give different growth performance and yield. This is made worst when the taxonomic revision of *Curculigo* was not recent and new varieties or species can occur (unpublished data) which have different capability of adaptation to cultivation. Secondly, on the note of adaptation, plants generally have different growth performance when being transferred from their original ecological site into cultivation, and plants from different population with different ecological environment will also show variations in growth performance. It is considered ideal to initiate cultivation using genetically similar and uniform plants obtained from the same origin. Therefore, prior to cultivation, it is prudent to look at the fundamental knowledge of the propagation of this plant. Attempts on propagation of lemba using seeds are time consuming due to poor seed germination and the plant propagation through underground corm and rhizomes produced a few number of plants [14]. Propagation for mass production of genetically uniform seedlings is achievable through aseptic *in vitro* cultures and is an advantage especially for plants which are highly valuable and which are difficult to propagate through conventional technique.

Tissue culture protocol for *Curculigo* was found for the species *C. orchioides* found as an endangered species in India [13, 15–18]. Report on tissue culture for *C. latifolia* is limited to one by Lim-Ho [19]. However, the emphasis of different plant react differently in tissue culture system has to be put into consideration especially when the number of species or varieties is inconclusive in Malaysia.

Thidiazuron (TDZ) is a phenyl urea-type plant growth regulator, which was mainly used as a cotton defoliant [20]. Thidiazuron is one of several substituted ureas that have been investigated for cytokinin activity [21]. In various cytokinin bioassays, TDZ exhibited strong cytokinin-like activity similar to that of N6-substituted adenine derivatives for using in plants tissue culture [22, 23]. The efficient effect of TDZ on *in vitro* cultures has been reported in many species for axillary shoot emersion and adventitious shoot regeneration [21, 24–26]. TDZ was reported to be more successful than BA and KIN for shoot regeneration in meadowsweet [24] and was also reported to play a major and distinctive role on adventitious shoot regeneration than purine-type cytokinins in carnation and rose [27]. It was also indicated that TDZ was effective than BAP on shoot regeneration in all hypocotyls, cotyledon, and stem explants of henbane [25].

Auxins are a class of phytohormones used for rooting in *in vitro* cultures. IBA is often the desirable auxin for rooting purposes. IBA was found to be the most favourable root inducer compared to IAA and NAA [28]. IAA is a natural auxin which is unstable comparing to synthetic auxins [29]. IBA is a more easily metabolized auxin with a slow release effect, whereas NAA, a highly stable auxin, remains in the tissues in free form and blocks root emergence [28, 30].

In relation to studies on the cultivation of *C. latifolia* in Universiti Putra Malaysia, the mass number of uniform plantlets was needed and tissue culture was employed. The objective of this study was to regenerate shoots from shoot tip cultures of *C. latifolia* using thidiazuron and indole-3-butyric acid and to induce root from regenerated shoots using different concentration of IBA.

## 2. Materials and Methods

### 2.1. Plant Material and Explants Source

Plant samples were collected from Branang, Negri Sembilan, Malaysia; replanted in polybags containing peat, soil, and sand (2 : 1 : 1, v/v); and maintained under 70% shade for at least six months. The shoot tips were collected and washed under running tap water and teepol for 35 minutes and then surface-sterilized by dipping in 70% ethanol for 1 minute and 30 seconds and 30% Clorox for 15 minutes. Then, the explants were pretreated with bavistin, chloramphenicol, ascorbic acid, citric acid, and PVP (0.1%) for nine hours on shaker (150 rpm). After pretreating explants were sterilised with 70% ethanol for one minute and 30 seconds and Clorox (30%) for 15 minutes followed by disinfecting with 0.1% (w/v) mercuric chloride for five minutes. Finally explants were cut into approximately 1 cm^2^ base and 4 mm height cut off, dipped in 10% Clorox for 10 minutes, and rinsed four times with sterile distilled water [31].

### 2.2. Culture Medium and Conditions

Surface sterilized shoot tips were placed onto MS basal salts and vitamins (SIGMA-ALDRICH) [32] supplemented with various concentrations of plant growth regulators, 3% (w/v) sucrose, and 0.2 (w/v) gelrite. The pH was adjusted to 5.8 prior to autoclaving at 121°C for 15 minutes at 15 p.s.i.

### 2.3. Shoot Regeneration

The basal medium was supplemented with different concentrations and combinations of thidiazuron (TDZ: 0, 0.5, 1, 1.5, and 2 mg L^−1^) and indole-3-butyric acid (IBA: 0, 0.25, and 0.5 mg L^−1^) for shoot regeneration and scalp induction. The cultures were incubated at 25 ± 2°C under dark condition for two weeks, followed by 16/8 h photoperiod (light/dark) and the light intensity of 40 *μ*mol m^−2^ s^−1^ provided by cool white fluorescent lamps during shoot regeneration and scalp induction. Explants were subcultured at four weeks intervals and data were recorded after 10 and 14 weeks of culture.

### 2.4. Root Induction

*In vitro* shoots (>2.5 cm) regenerated from regeneration experiment were cultured into the MS medium with different concentrations of IBA (0, 0.25, 0.5, and 0.75 mg L^−1^). Cultures were transferred onto new medium with the same constituents in four-week intervals for two times. All rooting cultures were kept under the 16/8 h photoperiod (light/dark) and the light intensity of 40 *μ*mol m^−2^ s^−1^ provided by cool white fluorescent lamps. Data were recorded after four and eight weeks of transferring into rooting medium.

### 2.5. Experimental Design and Data Analysis

The regeneration experiment was arranged in 3 × 5 factorial based on completely randomized design (CRD) with three replications with each replicates consisted of six flasks. One explant was cultured in one flask. The rooting experiment used a completely randomized design in three replications with five shoots per replication. Data were analyzed by analysis of variance (ANOVA) using SAS computer package (SAS institute Inc. 2005). Duncan's new multiple range test (DNMRT) was used for comparison among treatment means.

## 3. Results

### 3.1. Effects of TDZ and IBA on Shoot Regeneration from Shoot Tip

**Figure 1:**
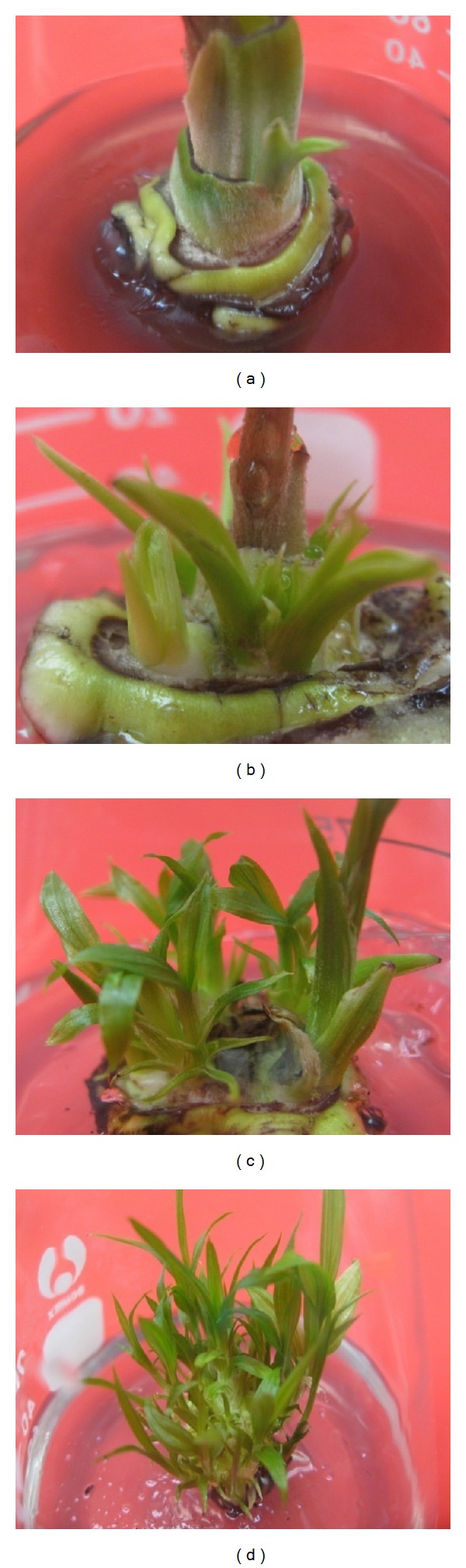
*In vitro *propagation of *C. latifolia.* (a) Shoot meristem and lateral bud grown after four weeks on MS medium supplemented with T0.5I0.25; (b) adventitious shoots have emerged after eight weeks; (c) development of shoots from shoot tip after 10 and (d) 14 weeks of culture.

Shoot tips incubated on MS medium supplemented with various concentrations of TDZ (0–2 mg L^−1^) and IBA (0–0.5 mg L^−1^) were able to regenerate shoots of *C. latifolia*. Cultures of explants on MS medium devoid of plant growth regulators (control) caused meristem elongation and development of a single plantlet and subsequently roots were initiated after two subcultures. In contrast, using TDZ prevented developing of elongated meristem to a single plantlet and caused axillary bud emersion and subsequently adventitious shoot induction (Figure 1). The meristematic shoot tips elongated over two weeks and axillary buds emerged from the leaf axils after four weeks of inoculation (Figure 1(a)). Axillary shoots increased in height and adventitious shoots began to regenerate after eight weeks of culture (Figure 1(b)). Initially, shoot induction was very slow, but after 10 weeks of culture new shoots were regenerated and shoots also grew on low concentration of growth regulators (Figure 1(c)). Regenerated shoots developed after 14 weeks of culture (Figure 1(d)).

The results of analysis of variance on shoot regeneration indicated that the percentage of explants producing shoots, number of shoots, and the shoot length were significantly influenced by the single application of TDZ and the interaction of TDZ and IBA. However, only the percentage of explants producing shoots was not affected by application of IBA alone. All the various combinations of TDZ and IBA were able to regenerate shoots from shoot tip explants. However the most effective concentration was combination of 0.5 mg L^−1^ TDZ and 0.25 IBA mg L^−1^, which induced an average of 3.08 and 7.52 shoots in 77.8 and 83.3% of cultured explants after 10 and 14 weeks of culture, respectively. Compared with media supplemented with other concentrations of TDZ and IBA, supplementation with this combination increased not only the percentage of explants producing shoots and average number of shoots, but also the average shoot length (Table 1). Explants incubated on medium supplemented with single application of IBA were not able to regenerate shoots. However, roots were initiated from explants on medium with the 0.25 and 0.5 mg L^−1^ IBA after eight weeks of culture.

### 3.2. Effect of TDZ and IBA on Scalp Induction from Shoot Tip

**Figure 2:**
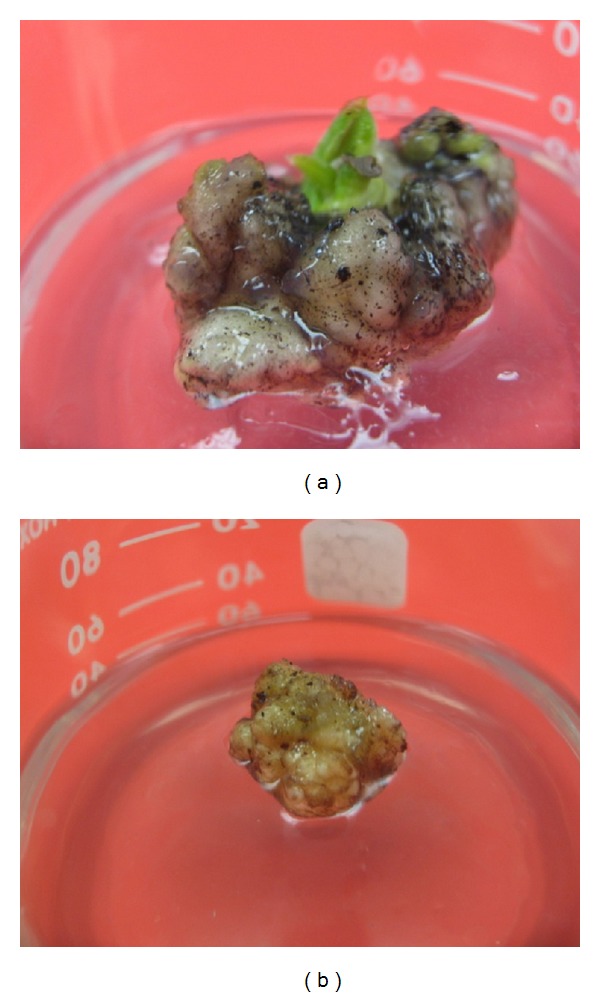
*In vitro *scalp induction of *C. latifolia*. (a) Scalp induction from shoot tip base on MS medium supplemented with 2 mg L^−1^ TDZ after 10 weeks; (b) yellowish scalp after segregation from explants and subculturing.

Six weeks after culturing, scalp was observed from the basal end of developing shoot tips. Whitish cluster (Figure 2(a)) regenerated from developing shoot tip directly without any visible interposing callus phase. The results of analysis of variance on percentage of explants produced scalp and scalp size showed that these parameters were significantly influenced by the simple application of TDZ. However, they were not affected by application of IBA alone and only scalp size was significantly affected by the interaction of TDZ and IBA. Although scalp was induced from all the treatments containing TDZ, it is evident that higher concentrations of TDZ (2 mg L^−1^) alone significantly increased the percentage of scalp induction (77.8%) and scalp size in both 10 and 14 weeks of culture (4.15 and 7.80 cm^2^ resp.) (Table 2). In contrast, with lower level of TDZ and IBA shoots induction was more prominent. However in some cases scalp was also induced at the base of explants. Scalps increased in size and their colour changed from whitish to yellowish after segregation from explants (Figure 2(b)).

### 3.3. Rooting

**Figure 3:**
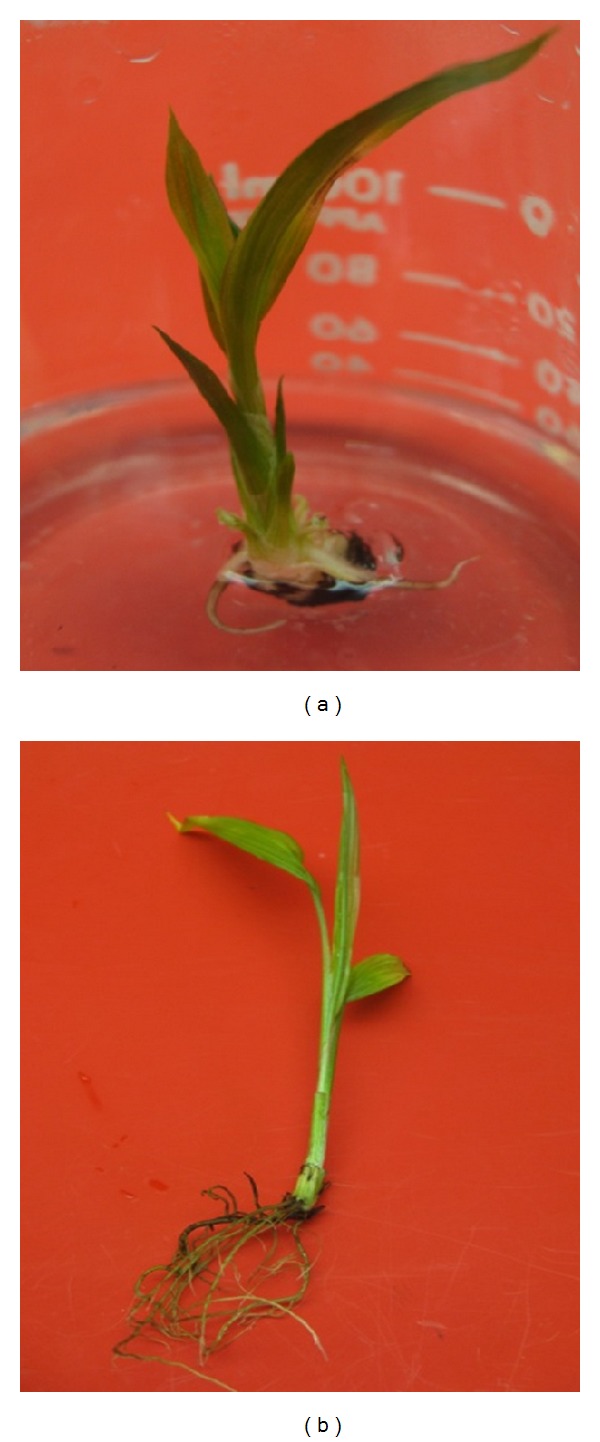
*In vitro* rooting of *C. latifolia*. (a) Emerged root form shoot after three weeks of transferring into MS free media and (b) a target plantlet with developed adventitious roots.

Although all the treatments applied in this study induced roots, the results differed depending upon the concentration of IBA used. Initial root induction at third week after the transfer of shoots was much quicker in the rooting medium free of growth regulators (Figure 3(a)). Induced roots elongated in length and lateral roots emerged from the primary roots and roots finally expanded well into the medium (Figure 3(b)). The results showed that there were no significant effects among treatments for data on percentage of shoots producing roots after four weeks of transferring onto rooting medium. However, the effects of different concentration of IBA on root number and root length after four weeks of transferring were significant at *P* < 0.05 and *P* < 0.01, respectively. However, after eight weeks of transferring to the rooting medium, the effects of the given treatments were highly significant for all parameters (Table 3). In an attempt to optimize the concentration of IBA in root formation and development, the result showed that roots were induced faster in MS medium devoid of plant growth regulators in comparison to other treatments. Furthermore, the highest percentage of shoots produced root (86.7%) and longest roots (4.87 cm) obtained in this treatment, whereas the maximum mean number of roots (7.64) was achieved at 0.25 mg L^−1^ IBA. This indicated that auxin is not necessary for *in vitro* root induction for *C. latifolia.*

## 4. Discussion

The plant regeneration system *via* axillary shoot emersion and direct shoot regeneration was established in this study. TDZ has been reported to play an important role in exogenously overcoming apical dominance, promoting lateral bud break and adventitious shoot induction in plant tissue culture [21]. In this study the most suitable concentration of TDZ for shoot regeneration from shoot tip explants was 0.5 mg L^−1^, which resulted in high percentage of shoot formation, producing high number of shoots per explant and shoot height. At this concentration of TDZ (0.5 mg L^−1^), its combination with 0.25 mg L^−1^ IBA was the best treatment as indicated by all recorded parameters.

Combined effects of cytokinin and auxin proved to be useful in shoot organogenesis in *Curculigo orchioides* [33], *Pinus massoniana* [34], and *Embelia ribes* [35]. In the present study the combination of 0.5 mg L^−1^ of TDZ and 0.25 mg L^−1^ of IBA resulted in both axillary shoot emersion and adventitious shoot regeneration. Similarly, it was also demonstrated that low concentration of TDZ could stimulate axillary shoot proliferation, whereas higher concentration could result in both axillary and adventitious shoot induction [21, 36].

This was also consistent with findings on potential applications of TDZ in adventitious shoot regeneration and synergistic effect of TDZ with auxins on inducing higher number of shoots [21, 24, 37].

TDZ, an integral component in tissue culture of woody plant as well as herbaceous crop species [21], is an efficient cytokinin in regeneration from shoot tip culture. Diverse crop species ranging from tropical fruit trees to root and tuber crops have been regenerated via TDZ-induced shoot production [36]. Multiple shoots were regenerated from hypocotyl and cotyledon explants on MS medium supplemented with a combination of 0.91 *μ*M of TDZ and 0.98 *μ*M of IBA with the highest number of shoots in hypocotyl explants rather than combination of kinetin and BAP with IBA in *Plantago afra* [38].

Although in the present study all concentrations and combinations of TDZ and IBA were able to regenerate shoots, the rapidly regenerated shoots from shoot tip explants, a high percentage of explants producing shoots, the higher number and length of shoots were observed in shoot tips cultured on media with the lowest concentration of TDZ and IBA. Similarly Yildirim and Turker [24] had also reported on shoot regeneration with lower concentrations of TDZ in *Filipendula ulmaria*. Likewise, Singh et al. [39] reported that low concentrations of TDZ (0.05–1.0 *μ*M) induced multiple shoots in pigeonpea. In another study, Peddaboina et al. [26] stated that using TDZ at a concentration of 4.54 *μ*M showed higher number of regenerated shoots in shoot tip explants of four *Capsicum* species. In contrast maximum mean number of shoots with higher percentage of multiple shoot regeneration was obtained when 2.5 *μ*M of TDZ was used in cotyledonary node explants of *Cassia sophera* [40]. 

Although the highest mean shoot length was obtained using 0.5 mg L^−1^ of TDZ in combination with 0.25 mg L^−1^ of IBA, but results showed that average shoot length was decreased with increasing concentration of TDZ and IBA. This occurrence is similar to reports on micropropagation of* Cassia sophera* using cotyledonary node explants where shoot length was increased with higher TDZ levels of upto 2.5 *μ*M, after which the shoot length began to decrease [40]. The shoot length reduction was observed when the concentration of IBA increased, even with the same level of TDZ used. Likewise Thomas [33] reported that the shoot length in *Curculigo orchioides* was reduced from 1.3 cm to 0.8 cm when a combination of 6 *μ*M of TDZ with 0.5 *μ*M NAA was increased to 6 *μ*M of TDZ with 1 *μ*M of NAA.

Reduction of recorded characteristics with further increase in TDZ concentrations was consistence with Thomas [33] observation who reported that a high level of TDZ in combination with auxin produced a negative effect in *Curculigo orchioides* shoot height. However, there was an increase on percentage of explants producing shoots and shoot numbers with 1.5 mg L^−1^ of TDZ at all levels of IBA (Table 1).

Scalps, a cauliflower-like structure, are actively proliferating meristems, derived from shoot tip culture and appear as a white bulbous structure and have been reported by several researchers in monocot plants, especially banana [41]. It was also reported in *C. orchioides* as a bulbil or bulblet [15, 16]. Rhizomes and corm are the underground vegetative propagable tissues of this plant. In *in vitro* cultures TDZ can stimulate a modified form of these tissues called scalp from the base of shoot tips which was the origin of corm and rhizomes in plant.

Cytokinins are vitally required for scalp induction in shoot tip cultures [42]. Sadik et al. [42] indicated authority of TDZ over BAP on scalp formation in banana. The higher cytokinin activity of TDZ was attributed to its ability to accumulate in cultured tissue to act as endogenous cytokinins [21]. The combination of high level of a cytokinin and very low level of auxin promotes scalp formation at the base of shoot tip explants [43, 44]. However, in the present study, maximum scalp induction was obtained from the single application of 2 mg L^−1^ of TDZ than in the combination of 2 mg L^−1^ of TDZ with IBA. On the other hand, combination of 2 mg L^−1^ of TDZ and 0.5 mg L^−1^ of IBA produced callus in some cultures. Since a high concentration of auxin is needed for callus induction more than scalp induction and TDZ alone can stimulate scalp induction, the response could be referred to as a mimicking effect of both auxin and cytokinin by TDZ on growth and differentiation of cultured explants, although structurally TDZ is different from auxins or purine-based cytokinins [36]. Thidiazuron is also well known as strong plant growth regulators for indirect regeneration of somatic embryogenesis [45, 46] and when scalp was formed on media supplemented with TDZ, this suggests that TDZ can also cause indirect regeneration of *C. latifolia*.

In the attempt to optimize the concentration of IBA in root formation and development, the results showed that roots were induced faster in MS medium devoid of plant growth regulators compared to other treatments. Furthermore, the highest percentage of shoots producing roots and the longest roots were obtained in this treatment, whereas maximum mean number of roots was achieved with 0.25 mg L^−1^ of IBA. Indigenous auxins in regenerated shoots are present in dissimilar amounts in different plant species and different plants need different amounts of auxin for root induction. Therefore plants require different amount of exogenous auxins for adventitious root formation. Although in *C. orchioides* root formation occurred in medium free of plant growth regulators [13, 17], the rooting was much lower without any auxin supplementation when compared to the present study. In previous studies on *C. orchioides* rooting reduced at supraoptimal concentration of IBA [13, 17, 33] as well. This suggests that auxin is not necessary for *in vitro* root induction in *C. latifolia.* However, initiation of lateral roots and more adventitious roots was stimulated by application of 0.25 mg L^−1^ of IBA. Stimulation of lateral roots by application of auxins has been reported in both monocot and dicot plants [47]. Further application of IBA up to 0.75 mg L^−1^ decreased the percentage of root induction, root numbers, and root lengths significantly. This observation is parallel to the report of Thomas [33], whereby a higher level of IBA produced a negative effect resulting in reduced percentage of shoots producing root, root number, and root length. Similarly, Yildirim and Turker [24] also stated that application of 2.46 *μ*M IBA effectively increased percentage of shoots producing roots and number of roots, while higher than optimal concentrations of IBA exerted subtractive effects on both parameters in *Filipendula ulmaria.*

Shoots placed on MS basal medium without auxin (control) produced the longest roots. It was also observed that the addition of IBA was unable to increase root length; and a decrease in root length was observed with an increase in IBA concentration up to 0.75 mg L^−1^ of IBA. This could be referred to the requirements of auxins at initial stage of root induction not at root development phase [48]. The inhibitory effects of exogenous auxins were also reported on root growth and elongation in monocots and dicots [47, 49]. Therefore, there exists an upper threshold for auxin to inhibit root initiation and elongation.

## 5. Conclusion

An efficient protocol for direct shoot regeneration and indirect scalp induction from *C. latifolia *shoot tip explants was developed. The preferred medium for shoot regeneration and scalp induction was MS medium supplemented with combination of 0.5 mg L^−1^ TDZ and 0.25 mg L^−1^ IBA and 2 mg L^−1^ TDZ alone, respectively. The induced shoots were easily elongated and rooted in MS medium devoid of plant growth regulators. However, more numbers of roots were induced from medium supplemented with 0.25 mg L^−1^ IBA. This report on micropropagation of *C. latifolia* is important for mass production plantlets for future cultivation. Moreover, this procedure can provide plant material for further physiological, biochemical, pharmacological, and genetic studies. On the note of obtaining genetically uniform plantlets, genetic analysis was carried out later in the studies but reported separately.

## Figures and Tables

**Table 1:** Effect of different concentration of TDZ and IBA on percentage of explants producing shoots, shoot numbers, and shoot length after 10 and 14 weeks of culture.

TDZ (mgL^−1^)	IBA (mgL^−1^)	Mean					
		Week 10			Week 14		
		Explants producing shoots (%)	Number of shoots	Shoot length (cm)	Explants producing shoots (%)	Number of shoots	Shoot length (cm)
0	0	—	—	—	—	—	—
0	0.25	—	—	—	—	—	—
0	0.5	—	—	—	—	—	—
0.5	0	38.89^cd^	3.00^ab^	1.01^bc^	61.11^bc^	5.44^b^	2.03^b^
0.5	0.25	77.78^a^	3.08^a^	1.43^a^	83.33^a^	7.52^a^	2.71^a^
0.5	0.5	61.11^b^	2.5^abc^	1.22^ab^	66.67^ab^	5.5^b^	1.98^bc^
1	0	33.33^d^	1.5^de^	1.12^ab^	50.00^bcd^	2.75^cd^	1.78^bcd^
1	0.25	38.89^cd^	1.83^cde^	1.17^ab^	44.44^cde^	3.28^c^	1.65^cde^
1	0.5	27.78^d^	1.33^ef^	1.00^bc^	38.89^def^	2.06^de^	1.47^def^
1.5	0	44.44^bcd^	2.61^abc^	1.09^abc^	50.00^bcd^	5.56^b^	1.84^bc^
1.5	0.25	38.89^cd^	2.28^abcd^	0.85^bc^	61.11^bc^	6.25^b^	1.71^bcde^
1.5	0.5	55.56^bc^	2.67^abc^	0.88^bc^	66.67^ab^	3.27^c^	1.26^fg^
2	0	33.33^d^	2.17^bcde^	0.74^cd^	38.89^def^	3.22^c^	1.40^ef^
2	0.25	11.11^e^	0.67^fg^	0.45^d^	27.78^ef^	1.67^e^	1.05^gh^
2	0.5	0.00^e^	0.00^g^	0.00^e^	22.22^f ^	1.67^e^	0.76^h^

*F*-test		∗∗	∗∗	∗∗	∗	∗∗	∗∗

ns: nonsignificant; *significant at *P* < 0.05; **significant at *P* < 0.01; —: no response.

**Table 2:** Effect of different concentration of TDZ and IBA on percentage of explants producing scalp and scalp size after 10 and 14 weeks.

TDZ (mgL^−1^)	IBA (mgL^−1^)	Mean			
		Week 10		Week 14	
		Explants producing scalp (%)	Scalp size (cm^2^)	Explants producing scalp (%)	Scalp size (cm^2^)
0	0	—	—	—	—
0	0.25	—	—	—	—
0	0.5	—	—	—	—
0.5	0	16.67^d^	1.36^de^	22.22^d^	1.88^g^
0.5	0.25	38.89^bc^	1.28^e^	38.89^bcd^	3.04^fg^
0.5	0.5	27.77^cd^	2.08^d^	33.33^cd^	3.89^ef^
1	0	55.56^ab^	3.47^bc^	55.56^abc^	4.76^de^
1	0.25	55.56^ab^	3.69^bc^	55.56^abc^	5.73^bcd^
1	0.5	50.00^b^	3.73^bc^	50.00^bc^	6.08^bcd^
1.5	0	55.56^ab^	3.83^bc^	55.56^abc^	6.31^bc^
1.5	0.25	61.11^ab^	4.74^a^	61.11^ab^	6.69^ab^
1.5	0.5	55.56^ab^	3.65^bc^	55.56^abc^	6.45^abc^
2	0	77.78^a^	4.15^ab^	77.78^a^	7.80^a^
2	0.25	55.56^ab^	3.17^c^	55.56^abc^	5.88^bcd^
2	0.5	44.44^bc^	2.12^d^	44.44^bc^	5.09^cde^

*F*-test		ns	∗∗	ns	∗∗

ns: nonsignificant; *significant at *P* < 0.05; **significant at *P* < 0.01; —: no response.

**Table 3:** Effect of different concentration of IBA on percentage of shoots producing root, number of roots, and root length after four and eight weeks of transferring onto rooting medium.

IBA (mgL^−1^)	Mean					
	Week four			Week eight		
	Shoot producing root (%)	Number of roots	Root length (cm)	Shoot producing root (%)	Number of roots	Root length (cm)
0	40.00^ab^	2.50^b^	3.15^a^	86.67^a^	4.48^b^	4.87^a^
0.25	46.67^a^	4.06^a^	0.91^b^	66.67^b^	7.64^a^	2.26^b^
0.5	26.67^b^	3.16^ab^	0.28^b^	53.33^b^	6.22^ab^	1.84^bc^
0.75	—	—	—	20.00^c^	1.33^c^	1.20^c^

*F*-test	ns	∗	∗∗	∗∗	∗∗	∗∗

ns: nonsignificant; *significant at *P* < 0.05; **significant at *P* < 0.01; —: no response.

Means followed by the same letter within a column are not significantly different according to the Duncan test at 5% level.

## Abbreviations

CRD: Completely randomized design
IBA: Indole-3-butyric acid
MS: Murashige and Skoog 1962 medium
PVP: Polyvinylpyrrolidone
TDZ: Thidiazuron.
